# Building sustainable operational research capacity in Pakistan: starting with tuberculosis and expanding to other public health problems

**DOI:** 10.1080/16549716.2018.1555215

**Published:** 2019-01-09

**Authors:** Razia Fatima, Aashifa Yaqoob, Ejaz Qadeer, Sven Gudmund Hinderaker, Einar Heldal, Rony Zachariah, Anthony D. Harries, Ajay M. V. Kumar

**Affiliations:** a Research Department, National TB Control Program, Islamabad, Pakistan; b Department of Hospital Management Information System, Pakistan Institute of Medical Sciences, Islamabad, Pakistan; c Centre for International Health, University of Bergen, Bergen, Norway; d Department of Tuberculosis, Blood Borne and Sexually Transmissible Infections, Norwegian Institute of Public Health, Oslo, Norway; e Special Programme for Research and Training in Tropical Diseases TDR, World Health Organization, Geneva, Switzerland; f International Union against Tuberculosis and Lung Disease, Paris, France; g Department of Clinical Research, London School of Hygiene & Tropical Medicine, London, UK; h International Union Against Tuberculosis and Lung Disease, South-East Asia Regional Office, New Delhi, India

**Keywords:** SORT-IT, operational research, capacity building, Pakistan, Tuberculosis

## Abstract

**Background**: For many years, operational research capacity has been a challenge and has remained a low priority for the health sector in Pakistan. Building research capacity for developing a critical mass of researchers in Pakistan was done through Structured Operational Research and Training Initiative (SORT IT) courses in Paris and Asia between 2010 and 2016.

**Objective**: The aim of this paper is to describe the journey of SORT-IT in Pakistan from its inception to progressive expansion and discuss the challenges and ways forward.

**Methods**: The journey began with the training of the Pakistan NTP research team lead in 2010 in an international SORT IT course at Paris. This was followed by training of two team members in Asia SORT IT courses in 2014 and 2015. These three then worked together to conceive and implement the first national Pakistan SORT IT course supported by WHO/TDR and the Global Fund in 2016. This was facilitated by international facilitators and local trained SORT-IT participants from Paris and Asia. This was followed by two further national SORT IT courses in 2017 and 2018.

**Results**: Between 2010 and 2017, a total of 34 participants from Pakistan had been enrolled in national and international SORT IT courses. Of the 23 participants from completed courses, 18(78%) successfully completed the course. In total 18 papers were submitted and up until June 2018, 15(83%) have been published and 21 institutions in Pakistan involved with operational research as a result of the SORT IT initiative.

**Conclusions**: The SORT IT course has been an effective way to build operational research capacity at national level and this has resulted in a large number of published papers providing local evidence for decision making on TB and other disease control programmes. The experience from Pakistan should stimulate other countries to adopt the SORT-IT model.

## Background

Pakistan is the sixth most populous country in the world and has a large public healthcare sector. The country is currently facing many challenges that include a double burden of communicable and non-communicable diseases [1], an unregulated private sector to which many patients go including those with presumptive TB [2], limited funding available for public health services and a rapidly increasing population. As a result, the health outcomes in Pakistan lag behind global targets, suggesting that there is an urgent need to understand how to maximize the benefits obtained from national and international health investments in the country.

An important approach to foster such understanding is to promote good-quality operational research. Despite recent calls by the World Health Organization (WHO) to strengthen health research capacity in low- and middle-income countries [3,4], building and sustaining health research capacity has remained a challenge and has been of low priority in Pakistan [5]. Despite the recommendations for coordinated and relevant health research by the Pakistan Medical Research Council in 2001 [6], there has been little progress in this area. Medical universities remain the major source of research for health and most of the best-conducted research comes from a few universities, usually in the private sector. The Ministry of Health and its various departments operate largely ‘vertical’ programs (TB, Malaria and HIV/AIDS) and most research in these areas has focused around assessing the burden of disease. Limited research has been done on implementation of heath programmes and their operational challenges. This article describes the successful implementation of Pakistan’s SORT IT (Structured Operational Research and Training Initiative) from inception to scale up as well as the challenges and ways forward.

## The start of operational research capacity building in Pakistan

Research is a key strategic area and core component identified in Pakistan’s National strategic and operational (PC1) [7] plans as well as in the new WHO END TB strategy (pillar III) [8]. In 2009, a research unit at the National TB Programme (NTP) was developed and became fully functional with the aim of designing and conducting locally relevant operational research. In 2012, the Pakistan NTP research team chief was trained as a researcher in a PhD program that included operational research at one of the international SORT IT Courses in Paris 2010, where she learnt about the philosophy of sustainable operational research around national priorities with a view to improving programme performance (14–17). Following this training, several operational research projects were undertaken in Pakistan around missing TB cases, private sector engagement and active case finding [9–13]. She is leading operational research capacity building agenda (SORT-IT) from 2016 onward. In order to further build national research capacity and develop a critical mass of researchers, two more research team members (a biostatistician and a public health officer) from the Pakistan NTP were trained in South Asian SORT-IT courses in 2014 and 2015. The previous successful participants of national SORT-IT course 2016 & 2017 were also facilitating the next courses. Operational research capacity defined as to develop the practical skills researchers for conducting operational research to identify operational challenges and barrier of disease control program activities and develop analytical method of problem solving and generating the evidence.

## Implementation of national SORT – IT courses in Pakistan

**Figure 1.**
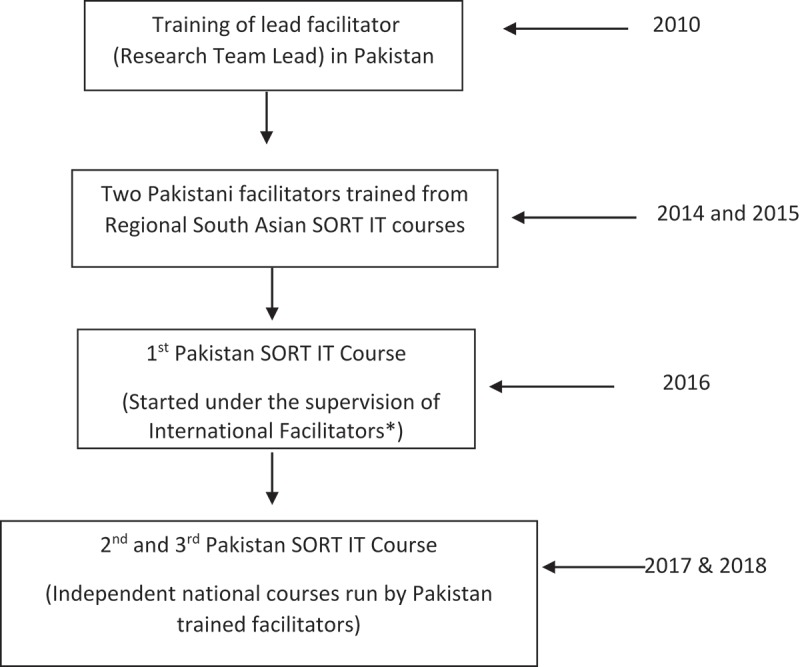
Flow of Pakistan SORT-IT courses from Inception. *The UNION and the University of Bergen

With this skill base developed, the NTP strived to secure resources to run its own national SORT-IT course in the country, and in 2016 the first course was implemented with joint funding from WHO-TDR (Special Programme for Research and Training in Tropical Diseases) and the Global Fund for AIDS, TB and Malaria. Subsequently, two further SORT IT courses were conducted in 2017 and 2018. The calls of the SORT IT course were widely disseminated in all health programs multiple ministries, academic and research organizations. The first course was facilitated by both international and national facilitators, while in subsequent courses it was just national faculty, many trained from the previous SORT IT courses, who shouldered the work of facilitation and mentoring. A description of how the Pakistan SORT IT Course was developed is shown in Figure 1.

## Selection of participants for national SORT IT courses

The selection of potential course participants was done using standard competitive selection criteria [14]. The course application requirements included submitting a brief research proposal with specific objectives and relevance to the applicant’s work, a letter of commitment from the participants and strong written support from their supervisors. The selection committee consisted of the technical advisor to the NTP, the research chief and experienced facilitators. In selecting participants, the committee considered criteria such as the public health degree, relevant experience, the feasibility of undertaking the research proposal and policy implications for Pakistan’s health systems. Eligible participants were health professionals working within national/provincial/or district programs (TB, Malaria, HIV/AIDs, and Hepatitis etc.), research institutions, non-governmental organisations (NGOs) and academia.

In the first cohort, the majority of the participants were selected from the TB control programme at national and provincial levels. In 2016, there were a total of 14 applications and this increased to 20 in 2017 and 42 in 2018. Eight participants were selected for the 2016 and 2017 courses and 10 were selected for the 2018 course. Two participants in 2016 and 2017 cohorts did not achieve the Milestone 3 as they did not get permission to publish their results from their affiliated institutions (Table 1). Language was not an issue in conducting the national Pakistan SORT-IT course in contrast to many other countries/settings.

**Table 1.** Details of Pakistan national SORT-IT course.

	2016	2017	2018
Total applicants	14	20	42
Selected participants for the course	8	8	10
Male female ratio	7:1	6:2	8:2
Successfully completed* (submitted papers)	6	6	Ongoing
Papers Published	6	4	Ongoing

*Achieved 4 milestones and submitted a paper

## Research capacity strengthening beyond TB

**Figure 2.**
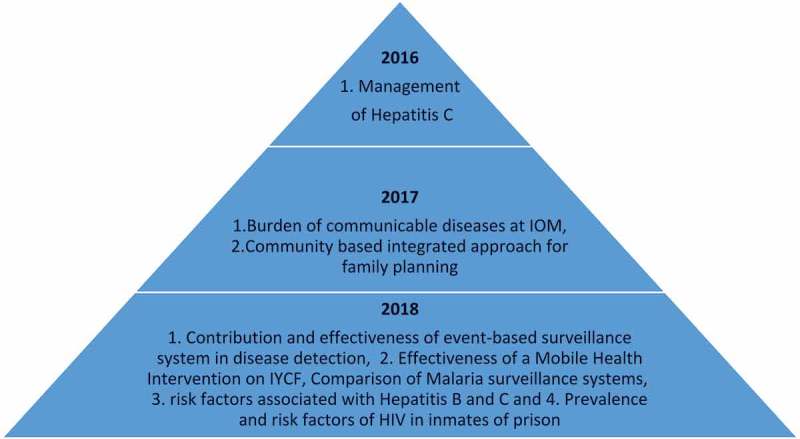
Expanding research subjects beyond Tuberculosis during three years of SORT IT courses in Pakistan. IOM: International Organization for Migration YCF: Infant and Young Child Feeding

In the first national SORT IT course (2016), there was only one non-TB theme. In the second course (2017), this increased to two non-TB themes and in the third course (2018), there were four non-TB themes. (Figure 2) Research capacity increased dramatically after the first national Pakistan SORT-IT course in 2016. Details of SORT-IT course outcomes are shown in Table 2. From the first training of Pakistani participants at SORT IT courses from 2010 to 2017, there have been a total of 34 participants from Pakistan enrolled in national and international SORT IT courses. Of the 23 participants from completed courses, 18 (78%) successfully completed the course. In total, 18 papers have been submitted and 15 (83%) have been published as of June 2018, in international peer reviewed index journals with impact factors ranging from 0.8 to 2.8 and the remainder are under review. All study areas were in Pakistan, almost all participants were from Pakistan, 68% of the last authors were from Pakistan and 36% were female, providing evidence that equity and gender balance are being promoted with respect to training female researchers in country. Currently, there are 21 institutions engaged within Pakistan in operational research through the SORT-IT courses. Table 3 illustrates some examples of published studies from SORT – IT courses and their effect on policy and practice from 2011 to June 2018.

**Table 2.** Research outcomes of participants who have attended in national and international SORT-IT courses (2010–2017).

Total number of participants enrolled	34
Total participants from completed courses	23
Number of participants successfully completed	18 (78%)
Total papers submitted (all SORT IT Pakistani participants)	18
– First author from Pakistan	100 %*
– Last author from Pakistan	68 %
– Female first authors	36 %
Papers published (up until June, 2018)	15 (83%)
No of institutions represented	21
Journal Impact factor (range)	0.80–2.8

*One of the participant was not a Pakistan national, but an expatriate working for MSF in Pakistan

**Table 3.** Examples of few SORT- IT Research Projects and their effect on policy and practice in Pakistan.

Project details	Key findings	Effect on policy and practices
Fatima R, Ejaz Q, Enarson DA, Bissell K. Comprehensiveness of primary services in the care of infectious tuberculosis patients in Rawalpindi, Pakistan. Public Health Action. 2011;1(1):13–15.	A cross sectional study was done to assess the initial loss to follow up in Rawalpindi districts, Pakistan. There were 16 145 suspects screened for TB and recorded in the laboratory registers. Of 1698 smear positive patients identified in the laboratory registers, 101 (6%) could not be identified in the treatment registers.	The article highlighted the need to strengthen hospital DOTS linkages as an intervention introduce to minimize loss of TB cases from tertiary care hospital linking various departments of hospitals as well as with the peripheral hospitals. Therefore, a phasic scale up of hospital dots linkages was established from 2011 onwards and further evaluated for increasing case notification especially in children in 2016 Pakistan SORT course ‘Mirza AS, Fatima R, Yaqoob A, Qadeer E, Wali A, Khurshid A, et al. Enhancing Childhood TB Notifications by Strengthening Linkages with Large Hospitals in Pakistan – Childhood TB in Large Hospitals, Pakistan. J Tuberc Res; 2018;6.’
Fatima R, Qadeer E, Yaqoob A, ul Haq M, Majumdar SS, Shewade HD, Stevens R, Creswell J, Mahmood N, Kumar AM. Extending ‘Contact Tracing’ into the Community within a 50-Metre Radius of an Index Tuberculosis Patient Using Xpert MTB/RIF in Urban, Pakistan: Did It Increase Case Detection? PloS one. 2016;11(11): 1–11.	In this study, an intervention was evaluated in which contact tracing was extended from smear positive TB cases household to 50 meter radius by using GIS technique. The overall yield of all forms TB patients among investigated was 22.3% among household and 19.1% in close community. The intervention contributed an increase of case detection of bacteriologically confirmed tuberculosis by 6.8% and all forms TB patients by 7.9%.	Same intervention was tested among DR TB patient through the support of Global Fund. Further scale up is planned in next Global fund applications.
Qadeer E, Fatima R, Haq MU, Yaqoob A, Kyaw NT, Shah S, Das M, Isaakidis P. Yield of facility-based verbal screening amongst household contacts of patients with multi-drug resistant tuberculosis in Pakistan. Journal of Clinical Tuberculosis and Other Mycobacterial Diseases. 2017;7:22–27.	In this cross sectional study, verbal screening was done amongst household contacts of patients with multi-drug resistant tuberculosis in Pakistan. Of total contacts, 56 (3.8%) were diagnosed with TB, among them 54(96%) with MDR-TB and 2(4%) with drug-susceptible TB.	The contact investigation for both sensitive and DR TB patient is given a special focus based on evidence generated and linked to the END TB strategy in revision to the national strategic plan.
Khurshid A, Hinderaker SG, Heldal E, Fatima R, Haq M, Yaqoob A, Ansari A, Anwar K, Qadeer E, Kumar AM. Did ‘Screeners’ Increase Pediatric Tuberculosis Case Notification in Sindh, Pakistan?. Journal of Tuberculosis Research. 2017;5(01):81–86.	To identify missing childhood Tuberculosis (TB) cases, ‘screeners’ (hospital-based health workers trained to screen accompanying contacts of TB patients for symptoms) were introduced in eight tertiary care hospitals of Sindh, Pakistan in 2013. There was a 55% increase in childhood TB notifications in 2014 compared to 2012 in facilities with screeners (n = 8) compared to 40% increase in facilities without screeners (n = 22). Screeners were not associated with increase in pediatric TB case notifications.	Based on evidence, the number of screeners are decreased in new funding request (NFR) grant 2017–20 by Private partners from Global Fund grants. Pediatrician training are conducting all over Pakistan which resulted in 4 times increased child TB notification.
Waheed Y, Khan MA, Fatima R, Yaqoob A, Mirza A, Qadeer E, Shakeel M, Heldal E, Kumar AM. Infection control in hospitals managing drug-resistant tuberculosis in Pakistan: how are we doing?. Public Health Action. 2017;7(1):26–31.	This was a descriptive study conducted between April and October 2016 with three components: 1) non-participant observation of service delivery areas (SDAs) (n = 82) in hospitals (n = 10) using structured checklists; 2) exit interviews with 100 patients (10 per hospital); and 3) interviews with 100 health-care workers (HCWs, 10/hospital). Of the 82 SDAs, posters were displayed in 34 (41%), mechanical ventilation was implemented in 79% and functional ultraviolet germicidal irradiation (UVGI) was available in only 26%. Patient interviews showed 50–65% adherence to triage and use of personal protective measures. Implementation of TB infection control measures in hospitals was suboptimal.	Retraining of HCWs and Improved supervision and monitoring of PMDT sides.
Safdar MA, Fatima R, Khan NM, Yaqoob A, Khurshid A, Haq MU, Wali A. Prevalence of Human Immune Deficiency among Registered Tuberculosis Patients across Pakistan during 2013–2015 – Prevalence of TB-HIV Co-Infection in Pakistan. Journal of Tuberculosis Research. 2018;6(01):96.	It was the cross sectional study assessing the prevalence of Human Immune Deficiency among Registered Tuberculosis Patients across Pakistan during 2013–2015. Among the screened TB patients 145 (0.66%) were found HIV reactive. The prevalence of HIV was higher (1.02%) in extra-pulmonary and male TB patients and B + ve. Only 113 (77.9%) reactive patients were found registered at ARV clinics for further treatment.	Sentinel sites for TB/HIV testing was scaled up from 17 to 40 across Pakistan. Introduction of TB-HIV Indicator in case finding reports (TB 07). Integration of TB-HIV services is in process.
Najmi H, Ahmed H, Halepota GM, Fatima R, Yaqoob A, Latif A, Ahmad W, Khursheed A. Community-based integrated approach to changing women’s family planning behaviour in Pakistan, 2014–2016. Public Health Action. 2018;8(2):85–90.	This study is the evaluation of community-based integrated approach to change the women’s family planning behaviour in Pakistan. Contraceptive prevalence rate was increased upto 10.7 % from the baseline (42.3% vs midline 53.0 %) with an increased in modern contraceptive rate 9.2%. Significant association was found between door-to-door counselling with the use of contraceptive methods, access to of public and private facilities for modern contraceptives. However, support group meeting and 24/7 helpline did not show any association with use of contraceptive method.	Based on the success of intervention, life skill based education modules were included to Secondary School Curriculum for Sind and intervention is scaled up in 10 districts.

## Discussion

Despite the establishment of many research institutions and a well-established academic infrastructure for the promotion of health research, this has remained a low priority area in the public health sector mainly because of lack of a research culture and/or demand for research in the country. The most critical deficiency in this area of health research is limited expertise in data analysis and reporting of research. Biostatisticians are almost non-existent and epidemiologists are in short supply. Lack of analytic skills is a major challenge and a large proportion of research remains unanalyzed. Poor scientific writing and reporting skills are another major hurdle in the dissemination of research findings in Pakistan [5]. To overcome these deficiencies, operational research training programs were introduced to enhance the capacity of data analysis and reporting skills to publish research findings in Pakistan. Through the Operational research capacity building, constraints/gaps in disease control programs can be identified and workable solutions can be suggested which can enhance the quality, coverage and performance of health system.

Developing research capacity to effectively understand and carry out health research is an integral component of the national strategic plan in Pakistan [7]. The process of embedding research in health care systems requires competent and skilled human resources and a supportive enabling environment. Since 2009, through the SORT IT initiative, Pakistan has started to train individuals involved in program monitoring and supervision and educate them to critically think about prioritizing operational research issues and conduct and publish programme-relevant operational research. The biggest challenge has been to develop the research environment and stimulate the interest of heath program directors and policy makers.

There were several challenges during the implementation of national SORT IT courses in Pakistan, of which securing funds and resources were major obstacles. The first course was supported by joint funding from WHO-TDR and The Global Fund with additional technical support from The Union, Paris, France and the University of Bergen, Norway. After its successful implementation, the next two courses were fully funded by The Global Fund (AIDS, Tuberculosis and Malaria). The Global Fund recognizes the key role of operational research in improving health programme performance and recommends an allocation of up to 10% of the total grants towards monitoring and evaluation including operational research [14]. Due to limited funding, national SORT IT courses have been run with federal level facilitators resulting in a high workload while more involvement of provincial trained facilitators could resolve this issue. The availability of high quality and skilled mentorship/facilitation is an ongoing challenge not only for Pakistan but elsewhere as well [15,16]. Women represent of low share of participants because of less women enrolment as public health professional in public health sector in Pakistan.

Despite this potential opportunity for programme strengthening, the implementation of operational research is still weak. Data sharing was the second obstacle encountered during the two courses for non-TB data from other programs. However, after the establishment of the common unit, a recently merged unit of TB, Malaria and HIV/AIDs, it has been possible to successfully broaden the engagement of health programmes/research/academic institutions other than TB.

A long-term funding mechanism is needed to continue and expand Pakistan’s national SORT IT courses to broaden research capacity within the country. In addition, the national operational research network generated through successful SORT-IT courses in Pakistan facilitates continued participation by provinces/regions and other programs to set out national research agendas according to public health priorities. SORT IT courses promote publications in peer-reviewed open access journals to enhance access for other low- and middle-income country (LMIC) researchers, but the costs for open access are as high as €1500–2500 in some journals, which can thus constitute a substantial part of the budget. Recommendations for reducing publication fees or making health research freely accessible to the user have been made before [17]. However, for Pakistan national courses, securing funds to cover publication costs remains a daunting challenge.

The key role of operational research in improving health programme performance is well recognised. The potential of current operational research is to explore further partnerships and networks with other programs and expanding the horizon to non-communicable diseases among different ministries are the future prospective of operational research.

## Conclusion

As identified in the WHO End TB strategy, research is the third important pillar and good-quality health cannot be achieved without research. We have found that a significant proportion of participants successfully completed a national SORT IT course in Pakistan and the trained participants continue to engage in research after the course as has happened elsewhere [18–20]. The subsequent engagement of multiple health programs and wide geographical coverage has also substantially increased in recent years. The current merger of three diseases in a common unit (HIV/AIDS, Tuberculosis and Malaria) under the Ministry of Health is another good opportunity to think of Integrated Research in the country. National SORT IT courses have shown encouraging findings and this initiative deserves to be sustained to address Pakistan health systems bottlenecks and programme implementations, thus contribute towards achieving universal health coverage.

## Paper context

The operational research capacity building has been a major challenge in a country therefore, it is important to describe the successful journey of Structured Operational Research and Training Initiative (SORT-IT) in Pakistan from inception to progressive expansion and discuss the challenges and the associated policy implication in Pakistan from various publications generated through this initiative. The experience from Pakistan course can be utilized as an example for other countries to adopt SORT-IT model.
